# Diversity of 26-locus Y-STR haplotypes in a Nepalese population sample: Isolation and drift in the Himalayas

**DOI:** 10.1016/j.forsciint.2006.05.007

**Published:** 2007-03-02

**Authors:** Emma J. Parkin, Thirsa Kraayenbrink, Jean Robert M.L. Opgenort, George L. van Driem, Nirmal Man Tuladhar, Peter de Knijff, Mark A. Jobling

**Affiliations:** a Department of Genetics, University of Leicester, University Road, Leicester LE1 7RH, UK; b MGC-Department of Human and Clinical Genetics, Leiden University Medical Centre, The Netherlands; c Himalayan Languages Project, Leiden University, The Netherlands; d Centre for Nepal and Asian Studies (CNAS) of Tribhuvan University (TU), Kirtipur, Nepal

**Keywords:** Y chromosome, STRs, Microsatellites, Haplotype, Nepal, Bhutan, Himalayas

## Abstract

Twenty-six Y-chromosomal short tandem repeat (STR) loci were amplified in a sample of 769 unrelated males from Nepal, using two multiplex polymerase chain reaction (PCR) assays. The 26 loci gave a discriminating power of 0.997, with 59% unique haplotypes, and the highest frequency haplotype occurring 12 times. We identified novel alleles at four loci, microvariants at a further two, and nine examples of *amelogenin-Y* deletions (1.2%). Comparison with a similarly sized Bhutanese sample typed with the same markers suggested histories of isolation and drift, with drift having a greater effect in Bhutan. Extended (11-locus) haplotypes for the Nepalese samples have been submitted to the Y-STR Haplotype Reference Database (YHRD).

## 1 Introduction

The analysis of multiple Y-chromosomal short tandem repeats (STRs) provides informative male-specific DNA profiles in forensic analysis. As well as possessing high discriminating power in distinguishing individuals, haplotypes defined by STRs can provide information about likely geographical origin, since they are often concentrated in particular populations or regions.

Population databases of Y haplotypes [1] are increasing in size and coverage, greatly contributing to the utility of Y-chromosomal analysis in forensic casework. In this study we describe alleles at 26 Y-STRs, and properties of the haplotypes they define, in a large sample of a previously unrepresented population, that of Nepal in the Himalayas. Eleven-locus haplotypes have been submitted to the Y-STR Haplotype Reference Database (YHRD), and full data are available from the authors on request. Our report follows guidelines for the publication of population data [2].

Sampling and Y-chromosomal analysis of 769 Nepalese males was undertaken as part of a larger collaborative project [3] investigating genetic diversity in Himalayan populations within the framework of their cultural and linguistic diversity [4]. Here we describe our initial findings with Y-STRs, treating the Nepalese sample as a single population; future publications will explore genetic relationships between subpopulations of the Himalayas. The sample represents 15 distinct ethnolinguistic groups widely distributed throughout Nepal, with ∼75% of sampled individuals speaking languages belonging to the Tibeto-Burman family, and the remainder speaking Indo-European languages.

In this study, we employ the same set of Y-STRs as that used recently to analyse 856 Bhutanese males [5]. This allows us to carry out a preliminary comparison of diversity and haplotype sharing between these two Himalayan samples.

## 2 Materials and methods

### 2.1 DNA samples

Seven hundred and sixty-nine Bhutanese males provided blood samples with informed consent, and DNA was extracted as described [3]. DNA samples from collections of the authors, including Y Chromosome Consortium (YCC) cell lines [6], were used as haplotype reference materials.

### 2.2 Y-STR multiplexes

Two PCR multiplexes (a 20plex [7] and a partially overlapping 14plex [5]) were used to type 26 Y-STRs, as follows: **DYS19**, **DYS385a/b**, DYS388, **DYS389I**, **DYS389II**, **DYS390**, **DYS391**, **DYS392**, **DYS393**, DYS425, DYS426, DYS434, DYS435, DYS436, DYS437, **DYS438**, **DYS439**, DYS447, DYS448, DYS460, DYS461, DYS462, YCAIIa/b, and Y-GATA-H4.1. The eleven Y-STR markers in the European ‘extended haplotype’ (http://www.yhrd.org/) are indicated in bold. The 14plex includes the amelogenin sex test. Full details of the protocol are given by Parkin et al. [5].

### 2.3 Y-STR nomenclature

Allele nomenclature (explained fully in Parkin et al. [5]) was according to Butler et al. [7] and Bosch et al. [8], with the exception of DYS439, DYS448 and Y-GATA-H4.1, where nomenclature was changed for compatibility with ISFG recommendations [9]. Compared to Butler et al. [7], seven repeats were subtracted from DYS439, three subtracted from DYS448, and eight added to Y-GATA-H4.1.

### 2.4 Calculations

Gene diversity and haplotype diversity were calculated using Arlequin [10]. A median-joining network was constructed using Network 4.0 ([11] http://www.fluxus-engineering.com/sharenet.htm), and the weighting scheme described by Qamar et al. [12].

## 3 Results and discussion

### 3.1 Diversity of alleles

Tables 1 and 2 show the allele frequency distributions for all the Y-STRs studied. Diversities of individual STRs are comparable with those observed in a recently studied Bhutanese sample: DYS385 (when considered as a genotype, Table 2) is the most diverse marker within the Y-STR set, with a gene diversity (*h*) of 0.915, and the most polymorphic single-locus marker is DYS439 (*h* = 0.726).

Previously unreported alleles (defined with reference to Butler [13], Parkin et al. [5] and STRBase, http://www.cstl.nist.gov/biotech/strbase/index.htm) were found at four loci, as follows: DYS426 (allele 13), DYS437 (allele 11), DYS439 (allele 15), DYS447 (alleles 17, 18 and 19).

‘Null’ alleles or multiple peaks were reproducibly obtained at a number of loci. For DYS448, three individuals carried null alleles, while one carried both alleles 20 and 21. For DYS461, one individual carried both alleles 13 and 14. As observed previously [5], DYS425 exhibits a relatively high frequency of various nulls and duplications.

Microvariants (partial alleles) were observed at two loci (Tables 1 and 2) and confirmed in uniplex assays after initial detection in multiplexes. Those at DYS385 were not investigated further, but those at DYS447 were analysed by sequencing, and shown to result from a deletion of 1 bp within the pentanucleotide repeat array [5].

### 3.2 *AMELY* deletion chromosomes

Nine chromosomes showed absence of the *amelogenin Y* (*AMELY*) peak in electropherograms. Analysis of sequence-tagged sites revealed that these chromosomes carry interstitial deletions of Yp including the *AMELY* locus (data not shown); none showed null Y-STR alleles, however, which is consistent with the size and location of known *AMELY* deletions with respect to the position of Y-STR loci [14]. A previous study has found *AMELY* deletions at a frequency of ∼2% in India [15], so our finding of deletions at 1.2% frequency in Nepal is not unexpected; in contrast, however, none were found in our previous study of Bhutan [5]. These *AMELY* deletion chromosomes form part of a large set that is currently being characterised, and will be described fully elsewhere.

### 3.3 Diversity of haplotypes

**Fig. 1:**
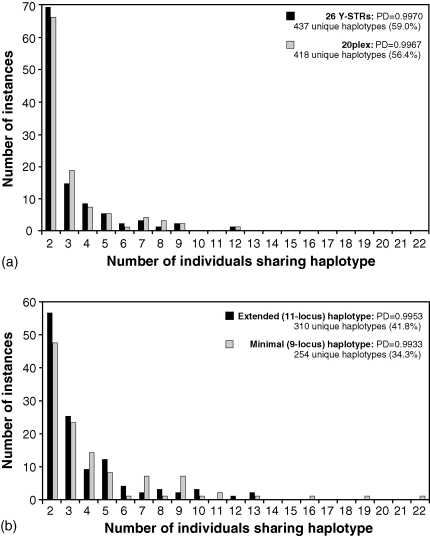
Haplotype diversity for (a) all 26 STRs and the 20plex, and (b) the extended and minimal haplotypes. Histograms show the frequency distributions of haplotypes present more than once in the dataset.

Haplotype diversity (equivalent to power of discrimination, PD) was calculated, omitting chromosomes carrying null alleles and duplications. This provided a sample size of 741. For the full set of 26 Y-STRs, there are 437 unique haplotypes (59.0%), and PD is 0.9970. The corresponding values for the 20plex [7], extended (11-locus) haplotype and minimal (9-locus) haplotype are shown in Fig. 1.

Fig. 1 also shows the distribution of haplotypes present more than once in the dataset. Despite the large number of loci used here, in the 741 males one 26-locus haplotype is shared by 12 individuals (Fig. 1a), and a further 13 haplotypes are shared by between 5 and 9 individuals; notably, all these common haplotypes are restricted to particular subpopulations, illustrating the influence of drift. Reduction to 11-locus extended haplotypes allows a global search within the YHRD (release 18): this fails to find matches for three of the six most common Nepalese extended haplotypes (frequency ≥10), consistent with isolation and drift.

### 3.4 Comparison of Y-STR datasets on Nepal and Bhutan

The availability of large Y-STR haplotype datasets on Nepalese and Bhutanese samples allows us to make comparisons between the frequencies and distributions of alleles and haplotypes in these two Himalayan populations.

Allele distributions at individual loci are similar between the Nepalese and Bhutanese samples, but this gives little information about population relationships. Particular rare and distinctive alleles may carry more information, because they probably reflect identity-by-descent: a good example of this is the sharing of microvariants at DYS447 [5], but apart from this there is little evidence for specific inter-population sharing.

**Fig. 2:**
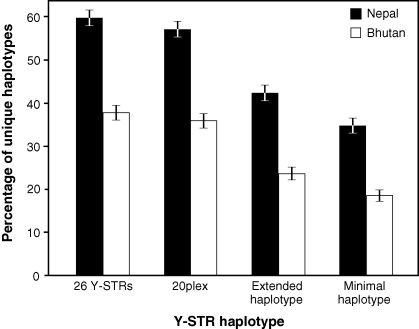
Percentage of unique haplotypes in Nepal compared to Bhutan. Haplotypes containing null or duplicated alleles are omitted, giving total sample sizes of 741 and 802, respectively. The error bars represent plus or minus one binomial standard error.

Comparison of haplotype distributions reveals a striking difference between the two populations. The proportion of unique haplotypes in the Nepalese sample is significantly greater than that in the Bhutanese, for all four haplotype resolutions considered (Fig. 2). For example, for the extended haplotype there are 41.8% (±1.8%) unique haplotypes in Nepal, but only 23.3% (±1.5%) in Bhutan. This is explained by the presence of several common haplotypes at high frequency in Bhutan: in the Nepalese dataset, the most common extended haplotypes are each present in 13 individuals, while in the Bhutanese there are haplotypes present in 15, 16, 24 (two instances) and 27 individuals [5].

**Fig. 3:**
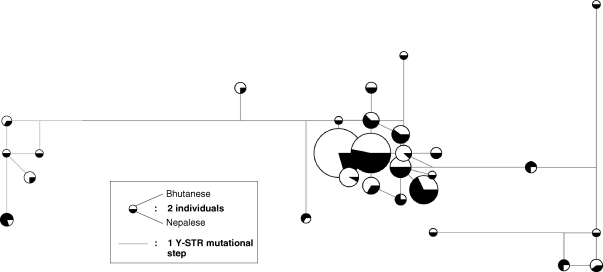
Median-joining network of haplotypes shared between Nepal and Bhutan. Note that the 40 shared extended haplotypes described in the text are reduced to 28 when the bilocal Y-STR DYS385 is removed for network construction. Circles represent Y-STR haplotypes (based on DYS19, DYS389I, DYS389II-I, DYS390, DYS391, DYS392, DYS393, DYS438, DYS439), with area proportional to number of instances. Lines represent Y-STR mutational steps.

There are no 26-locus haplotypes shared between Nepal and Bhutan, indicating an absence of very recent gene flow. However, forty extended haplotypes are shared between the two samples, and their relationships (omitting the bilocal marker DYS385) are illustrated in a median-joining network in Fig. 3. Most of them fall into one large cluster, with haplotypes linked by single mutational steps, probably representing a common Y-SNP haplogroup. Other shared haplotypes are more widely spread, and may represent several different haplogroups.

To ask if these shared extended haplotypes are more generally common and widespread, we sought matches for the six most predominant examples (combined frequency >10) within the YHRD. Three of the six haplotypes find a total of six exact matches, all within populations originating from China or the Indian subcontinent. We also find a total of 30 one-step mutational neighbours for five of the six haplotypes, all of Asian origin. One haplotype finds neither exact matches nor one-step neighbours. Thus, the common haplotypes shared between Nepal and Bhutan are Asian-specific, but not generally frequent.

### 3.5 Concluding remarks

Our study emphasises the discriminating power of high-resolution Y-STR typing, and provides the first substantial dataset on a Nepalese sample. The comparison of Nepalese and Bhutanese datasets reveals an interesting overall picture of isolation and drift within these Himalayan populations, with drift having a greater effect in Bhutan than Nepal. Haplotype sharing provides evidence of some gene flow between Nepal and Bhutan, or possibly of gene flow into both from some other population. Further light will be thrown on these relationships when Y-SNP data become available.

## Figures and Tables

**Table 1:** Frequencies of alleles at 22 of the 26 Y-STRs

Allele	19	388	390	391	392	393	425	426	434	435	436	437	438	439	447	448	460	461	462	389I	389II-I	H4.1
7					0.022								0.001				0.004					
8													0.003				0.001					
9				0.053									0.085				0.511	0.018				
10		0.563		0.776	0.072			0.003	0.087	0.001			0.168	0.151			0.270	0.008	0.001	0.003		
11		0.004		0.164	0.261	0.009	0.001	0.826	0.870	0.982	0.001	0.003	0.719	0.248			0.176	0.140	0.217	0.014		
12	0.001	0.289		0.005	0.059	0.675	0.948	0.170	0.016	0.017	0.978		0.025	0.390			0.038	0.645	0.684	0.524		
13	0.044	0.068		0.001	0.023	0.211	0.018	0.001	0.027		0.021	0.001		0.192				0.160	0.096	0.290	0.001	
14	0.644	0.020			0.497	0.100						0.386		0.016				0.027	0.001	0.165		
15	0.241	0.046			0.049	0.005						0.544		0.003						0.004	0.168	
16	0.066	0.008			0.016							0.065				0.003					0.550	
17	0.004	0.001			0.001							0.001			0.003	0.046					0.189	
18		0.003													0.003	0.025					0.087	0.022
19															0.005	0.242					0.005	0.280
20																0.614						0.586
21			0.005													0.064						0.104
22			0.051												0.057	0.003						0.008
23			0.490												0.501							
24			0.321												0.144							
25			0.124												0.086							
26			0.008												0.100							
27			0.001												0.072							
28															0.010							
29															0.004							
21.4															0.001							
22.4															0.013							
23.4															0.001							
9–12							0.001															
10–11							0.004															
11–12							0.005															
12–13							0.005															
13–14																		0.001				
20–21																0.001						
Null							0.017									0.004						

*h*^a^	0.521	0.593	0.639	0.368	0.673	0.490	0.040	0.289	0.235	0.036	0.043	0.551	0.447	0.726	0.702	0.553	0.633	0.536	0.476	0.614	0.626	0.567
*h*(Bh)^b^	0.604	0.518	0.569	0.421	0.546	0.442	0.187	0.244	0.244	0.046	0.092	0.553	0.452	0.713	0.663	0.590	0.679	0.434	0.363	0.592	0.504	0.598

a Calculation of gene diversity, *h*, excludes null alleles and duplications.

b Comparative Bhutanese values from [5].

**Table 2:** Frequencies of genotypes at DYS385 and YCAII

Genotype	DYS385	YCAII
10–14	0.005	
11–11	0.017	
11–12	0.007	
11–13	0.004	
11–14	0.070	
11–15	0.001	
11–16	0.001	
11–18	0.003	
11–19	0.001	
11–20	0.001	
12–12	0.004	
12–13	0.003	
12–14	0.009	
12–15	0.001	
12–16	0.027	
12–17	0.029	
12–18	0.014	
12–19	0.007	
12–20	0.012	
13–13	0.022	
13–14	0.022	
13–15	0.003	
13–16	0.027	
13–17	0.056	
13–18	0.182	
13–19	0.177	
13–20	0.060	
13–21	0.014	
13–22	0.001	
13–23	0.003	
14–14	0.004	
14–15	0.007	
14–16	0.010	
14–17	0.013	
14–18	0.044	
14–19	0.031	
14–20	0.027	
14–22	0.001	
15–15	0.004	
15–16	0.009	0.009
15–17	0.007	
15–18	0.013	
15–19	0.003	0.014
15–20	0.007	
15–21	0.001	
16–16	0.001	0.008
16–17	0.004	0.003
16–18	0.004	
16–19	0.003	0.010
16–20	0.003	
16–22		0.001
17–17	0.003	0.036
17–18		0.029
17–19		0.606
17–20		0.113
17–21		0.091
17–22		0.001
17–23		0.001
18–18		0.012
18–19	0.001	0.025
18–20		0.001
19–19	0.001	0.036
20–20		0.001
20–21		0.001
13–17.2	0.001	
13–18.2	0.016	

*h*	0.915	0.607
*h*(Bh)^a^	0.921	0.524

a Comparative Bhutanese values from [5].
